# Dimensionless squared jerk: An objective differential to assess experienced and novice probe movement in obstetric ultrasound

**DOI:** 10.1002/pd.5855

**Published:** 2020-11-05

**Authors:** Brian P Dromey, Shahanaz Ahmed, Francisco Vasconcelos, Evangelos Mazomenos, Yada Kunpalin, Sebastien Ourselin, Jan Deprest, Anna L David, Danail Stoyanov, Donald M Peebles

**Affiliations:** ^1^ Elizabeth Garrett Anderson Institute for Women's Health University College London London UK; ^2^ Wellcome/EPSRC Centre for Interventional and Surgical Sciences (WEISS) University College London UK; ^3^ School of Biomedical Engineering and Imaging Sciences King's College London London UK; ^4^ Department of Obstetrics and Gynecology University Hospitals Leuven Leuven Belgium; ^5^ NIHR University College London Hospitals Biomedical Research Centre London UK

## Abstract

**Objective:**

Widely accepted, validated and objective measures of ultrasound competency have not been established for clinical practice. Outcomes of training curricula are often based on arbitrary thresholds, such as the number of clinical cases completed. We aimed to define metrics against which competency could be measured.

**Method:**

We undertook a prospective, observational study of obstetric sonographers at a UK University Teaching Hospital. Participants were either experienced in fetal ultrasound (*n* = 10, >200 ultrasound examinations) or novice operators (*n* = 10, <25 ultrasound examinations). We recorded probe motion data during the performance of biometry on a commercially available mid‐trimester phantom.

**Results:**

We report that Dimensionless squared jerk, an assessment of deliberate hand movements, independent of movement duration, extent, spurious peaks and dimension differed significantly different between groups, 19.26 (SD 3.02) for experienced and 22.08 (SD 1.05, *p* = 0.01) for novice operators, respectively. Experienced operator performance, was associated with a shorter time to task completion of 176.46 s (SD 47.31) compared to 666.94 s (SD 490.36, *p* = 0.0004) for novice operators. Probe travel was also shorter for experienced operators 521.23 mm (SD 27.41) versus 2234.82 mm (SD 188.50, *p* = 0.007) when compared to novice operators.

**Conclusion:**

Our results represent progress toward an objective assessment of technical skill in obstetric ultrasound. Repeating this methodology in a clinical environment may develop insight into the generalisability of these findings into ultrasound education.

**What is already known about this topic?**

- There is little global consensus on how to train, assess and evaluate skill in obstetric ultrasound.
- Curricula, where present, are often based on clinical volume, rather than objective outcomes of competence.
- The acquisition of complex psychomotor tasks follows a learning curve, but they have not been objectively measured or classified.
- DSJ has been proposed as an objective parameter in endoscopic surgery where similar issues are faced.

**What does this study add?**

- Inspired by comparable training challenges in endoscopic surgery, this study observed operator behaviour by tracking the ultrasound probe during an ultrasound scan.
- We included experienced and novice operators.
- We describe measurable and reproducible performance differences between the groups.
- When time and DSJ are considered together, the experienced group had less unwanted and more purposeful movements.
- Our results show that quantifiable performance changes can be defined using objective measures.

## 1 INTRODUCTION

Ultrasound is a dynamic, real‐time imaging modality that is widely used in clinical obstetrics for screening of congenital anomalies, to monitor fetal growth and to assess fetal well‐being. The quality of an ultrasound examination is known to be operator dependent and to have high inter‐operator variability.^1^ This directly affects the information available to the clinician. Consequently, training and competence assessment are of great importance to ensure effective, reproducible and safe clinical practice. Varying standards for training and assessment of skills have been suggested by bodies such as the American Institute of Ultrasound in Medicine (AIUM)^2^ and The International Society of Ultrasound in Obstetrics and Gynaecology (ISUOG).^3^ National colleges have introduced varying requirements. The French National College of Gynaecologists and Obstetricians (CNGOF) require practitioners to pass a national examination to obtain certification. On the other hand in Sweden and Italy there is neither a formal curriculum in obstetric ultrasound, nor a minimum number of scans to complete prior to independent practice.^4^ Similar challenges for trainees have been reported in other medical^5^ and surgical^6^ specialities, where reduced training hours have led to concerns^7^ that current specialists‐in‐training will have had fewer training hours than their predecessors.^8^

Unlike first trimester ultrasound,^9^ a widely accepted threshold for competency and service audit has not been established for fetal biometry. Where defined, outcomes of training curricula are often based on arbitrary thresholds, such as the number of clinical cases completed rather than objective measures of competence.^10^ These thresholds assume competence based on the expectation that experience equates to expertise. This assumption has been challenged by Tolsgaard et al,^11^ which found that that some experienced ultrasound operators did not display expert‐like behaviours, despite daily use of obstetric ultrasound in their clinical practice. The authors hypothesised that inadequate basic training may be a root cause, as the operators did not have the correct foundation to benefit from later clinical training. The authors further hypothesised that the expected improvement in performance was not seen because sustained deliberate practice rarely occurs in clinical practice.

The acquisition of complex skills and psychomotor tasks follows a learning curve. Ultrasound examinations, much like in minimally invasive surgery (MIS) hand‐eye coordination, require the operator to interpret a dynamic image produced by the three‐dimensional (3D) position and motion of the ultrasound probe by means of a two‐dimensional (2D) visual display. The challenge of delivering high quality training leading to safe, reproducible practice has been considered extensively in minimally‐invasive surgery.^12^, ^13^ Obstetric ultrasound requires a detailed understanding of the capabilities and limitations of ultrasound equipment, as well as knowledge of normal maternal and fetal anatomy, as well as an appreciation of abnormal findings. The experienced operator will be able to combine this knowledge with the technical ability to manipulate transducer and display settings to optimise the image, leading to higher objective image quality. It is accepted that performance improves with training and experience,^7^, ^8^, ^14^ but this observation has not been objectively measured or classified.

Dimensionless squared jerk has been proposed as an objective parameter to discriminate between expert and novice operators in endoscopic surgery.^15^ Dimensionless squared jerk is a measure of deliberate hand movements and is a derivative of acceleration with respect to time and distance while remaining independent of spurious peaks and dimension within the movement.^16^ Dimensionless squared jerk is normalised with respect to time duration, so that only the shape of the trajectory ‐ and not its dimension/extension ‐ contributes to jerk. Dimensionless squared jerk quantifies common deviations from smooth, coordinated movement and it has been accepted as an objective parameter to quantify hand motion in different disciplines, such as parkinsonism, kinetics, and optometry.^17^, ^18^ To our knowledge, no study quantifying hand motion using Dimensionless squared jerk during simulated ultrasound has been performed. Inspired by comparable training challenges in endoscopic surgery, this study observed operator behaviour by tracking the ultrasound probe during an ultrasound scan and by comparing the images obtained. We hypothesise that dimensionless squared jerk could be used to differentiate experienced from novice operators. We compared the results between experienced and novice ultrasound operators with the aim of establishing specific, measurable and reproducible performance differences between experienced and novice operators.

## 2 METHODS

We undertook a prospective, observational study of medical practitioners who were accredited specialists or specialty trainees in Obstetrics & Gynaecology at University College London Hospital NHS Foundation Trust, London, UK (UCLH). Participants were empirically divided between either experienced in fetal ultrasound (*n* = 10, >200 fetal ultrasound examinations, “experienced”) or novice operators (*n* = 10, <25 ultrasound examinations, “novice”). These numbers were selected as The European Board and College of Obstetrics and Gynaecology (EBCOG) guideline on obstetric training recommends that trainees complete a logbook as part of their training which contains 200 obstetric ultrasound examinations.^19^ The study was undertaken at the Obstetric Ultrasound Unit at UCLH. Clinical experience varied from Foundation Year 2 Doctors to Consultants in Fetal Medicine. The study was exempt from review by the NHS Research Ethics Committee as it was not performed on patients. Informed consent was obtained from each participant.

**FIGURE 1:**
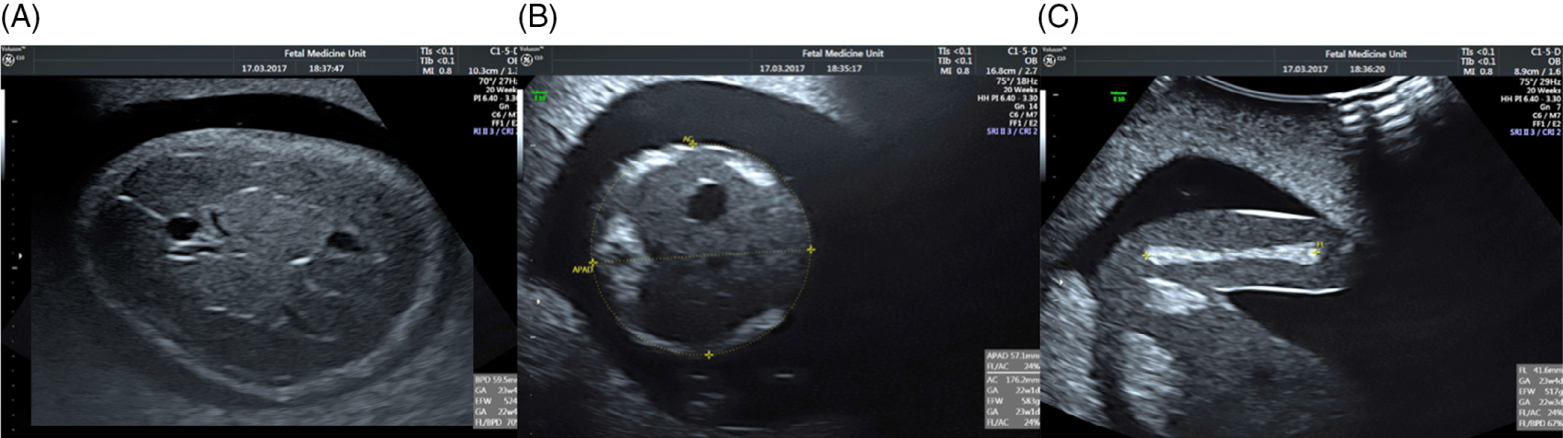
Images of the fetal anatomy in the second trimester phantom. Image (A) represents the Transventricular Plane, used for measurement of the Biparietal Diameter (BPD), Image (B) represents the Transabdominal Plane, used for measurement of the Abdominal Circumference (AC), Image (C) represents the required view of the Femur used to measure the length of the bone (FL) [Colour figure can be viewed at wileyonlinelibrary.com]

Each participant was asked to obtain standard 2D fetal measurements using a clinical GE Voluson E8 ultrasound machine (GE Healthcare, Chicago, Illinois) on a commercially available second trimester phantom (SPACE‐FAN ST, Kyoto Kagaku Co., Ltd, Kyoto, Japan). The phantom was chosen as it simulates the fetal skeleton and the key structures required to obtain technically adequate cross‐sectional images. The simulator consists of a fetal skeleton fixed within an oval shaped abdomen. The experimental set‐up is shown in Figure 1(A) and a detailed view of the sensor attached to the ultrasound probe is shown in Figure 1(B).

The required views were:

- The trans‐ventricular plane, with bi‐parietal diameter (BPD) measurement.
- The trans‐abdominal plane, on which the Anterior Posterior Abdominal Diameter (APAD), the Transverse Abdominal Diameter (TAD) and the Abdominal Circumference (AC) can be measured.
- A view of the femur and a measurement of its length (FL).

These views were chosen as these are the views are necessary to calculate fetal weight, in accordance with Hadlock B formula and are in accordance with guidance on the estimation of fetal weight from ISUOG.^20^ The Hadlock B formula was used to calculate the estimated fetal weight as it is widely used to estimate fetal weight and it has a lower error rate than other formulas.^21^

Prior to commencing the scan each participant was given written instructions (supplement 1), including images of the required planes, Figure 1, and up to 5 min to familiarise themselves with the operation of the ultrasound scanner. Both novice and expert operators were allowed unlimited time to complete the task. Participants were permitted to refer to the instructions at any time during the task, if they wished to.

**FIGURE 2:**
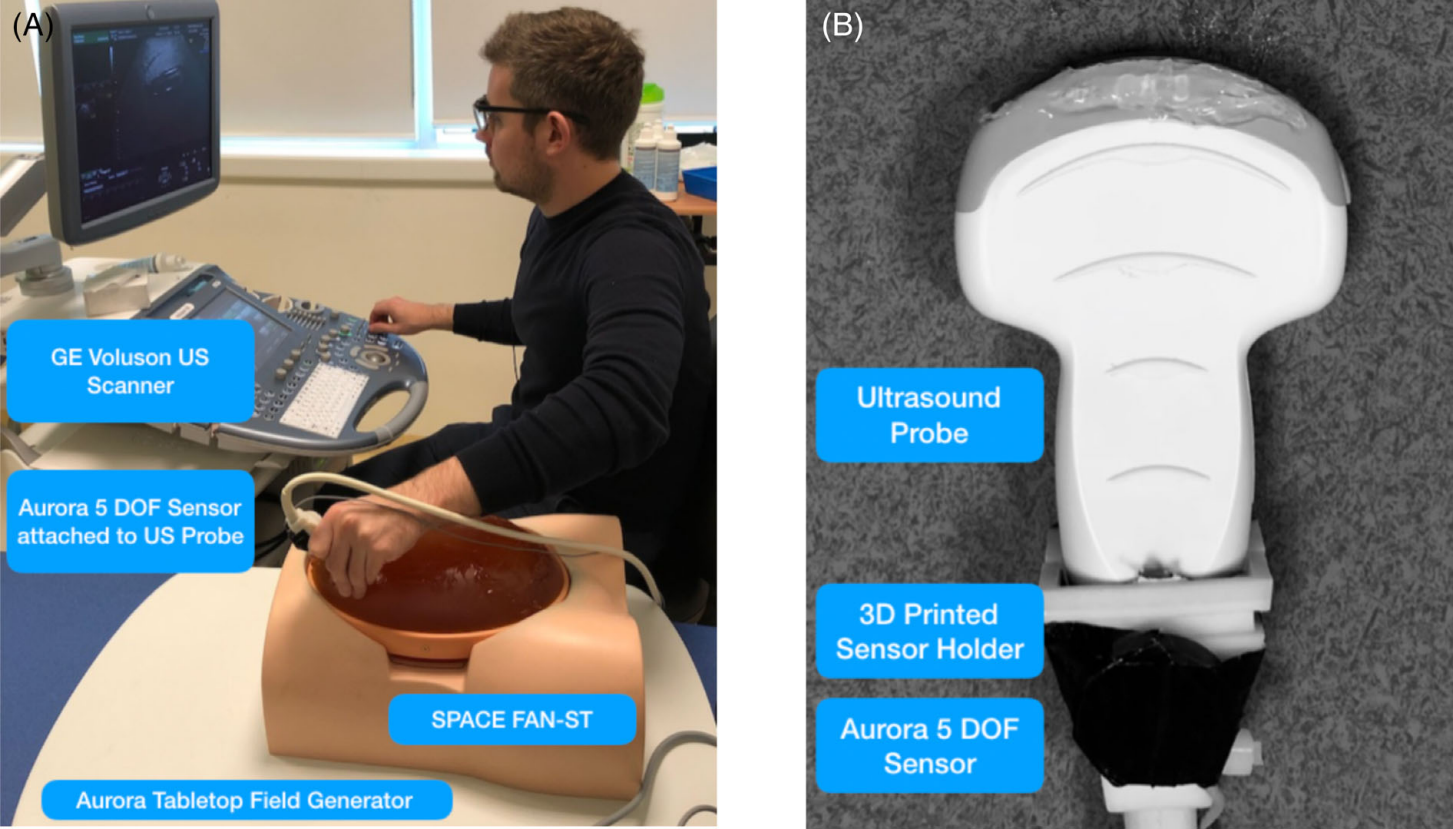
(A) The experimental set‐up, showing the phantom, ultrasound scanner and electromagnetic probe tracking system. (B) A Close‐up of the ultrasound probe, with an Aurora 5 DOF electromagnetic sensor attached using the 3D‐printed sensor holder [Colour figure can be viewed at wileyonlinelibrary.com]

The position of the probe was tracked using the Aurora electromagnetic tracking system (NDI Inc., Ontario, Canada). The experimental set‐up is shown in Figure 2. To track the probe a 6 degree‐of‐freedom (DOF) sensor was attached to the ultrasound probe by means of a custom 3‐D printed holder, as shown in Figure 1(B). The sensor used was 6DOF sensor, (Aurora Part Number: 610066), only translation (3 DOF) was used to compute dimensionless jerk and path length. The phantom was placed on an Aurora planar field generator on the movable couch used clinically to simulate the exact position of a patient, this is shown in Figure 1(A). This allowed the position of the probe to be recorded using NDI's “Aurora ToolBox” software. Each image was saved to the memory of the ultrasound scanner and later all captured images were transferred to the research database. The measurements (B.P.D., A.C. and F.L.) obtained by each participant were compared to the specifications provided by the manufacturer of the phantom, assumed to represent the ground truth. Two clinicians (B.D. and Y.K.) were asked to score the images individually for quality control in fetal biometry, using the scoring system described by Salomon et al.^22^

**Cephalic Plane (HC)**: One point was assigned for each of Symmetrical Plane, Visible Thalamus, Cavum Septi Pellucidum Visible, Absence of Cerebellum, Head occupying >30% of the Image (Zoom).

**Abdominal Circumference (AC)**: Symmetrical Plane, Stomach Bubble Visible, No Kidney Visible, Abdomen Occupying >30% of the image (Zoom), Callipers Placed Correctly.

**Femur Length (FL)**: Both ends of the bone visible, angle <45°, Femur Occupying >30% of the Image, Callipers placed correctly.

Both reviewers were blinded to the participants clinical experience or whether the images being scored were from the novice or expert groups. The maximum possible score was 15.

Dimensionless squared jerk (DSJ)^16^, ^23^ was calculated for each novice and expert participant, using the formula described by Hogan and Sternad.

$$\text{Jerk}\left(i\right)=\frac{\left(\int_{t1}^{t2}x⃛{\left(t\right)}^{2}\mathrm{dt}\right)D^{5}}{A^{2}},$$

where: *A* is movement amplitude or extent, *t*1 = initial time; *t*2 = final time; *t*2 − *t*1 = completion time; D = path length and *D* = *t*_2_ − *t*_1_ is duration.

## 3 RESULTS

Experienced operator performance was associated with a shorter time to task completion and probe travel. On average a novice operator took almost four times longer to perform the task. The mean experienced operator time to completion was 176.46 s (SD 47.31) while in novices it was 666.94 s (SD 490.36, *p* = 0.0004). In the novice group the ultrasound probe travelled a further distance on the phantom, on average 3.3 times as far 2234.82 mm SD 188.50, versus 521.23 mm SD 27.41, *p* = 0.007 for experienced operators.

When evaluating the images used to estimate fetal weight in accordance with the scoring system detailed in the methodology, we found that experienced operators achieved a higher quality image score (mean score 11.8, SD 1.87) than novice operators (10.2 SD 1.46, *p* = 0.04) (Table 1). We found that more experienced operators achieved a higher score for the submitted images, but this did not correlate with the accuracy of assessment of the AC or FL. The estimated fetal weight did not differ significantly between the groups 633 g SD 33.94 and 630 g SD 78.18, *p* = 0.93 for the experienced and novice groups, respectively.

**TABLE 1:** Mean biometry results, image results, estimated fetal weight, time to completion and path length for expert and novice operators

	Expert ± SD	Novice ± SD	*p*
BPD (mm)			
58 (Manufacturer Spec)	60.21 ± 1.54	62.59 ± 3.21	0.03
AC (mm)			
177 (Manufacturer Spec)	181.52 ± 4.05	183.12 ± 5.95	0.79
FL (mm)			
37 (Manufacturer Spec)	44.32 ± 1.87	44.04 ± 2.50	0.56
Estimated fetal weight			
515 g (Hadlock)*	633.3 ± 33.94	630.7 ± 78.18	0.93
Image score	11.8 ± 1.87	10.2 ± 1.46	0.04
Time (s)	176.46 ± 47.31	666.94 ± 490.36	0.0004
Probe path length (mm)	521.23 ± 27.41	2234.82 ± 188.50	0.007
Dimensionless Jerk	19.26 ± 3.02	22.08 ± 1.05	0.01

**FIGURE 3:**
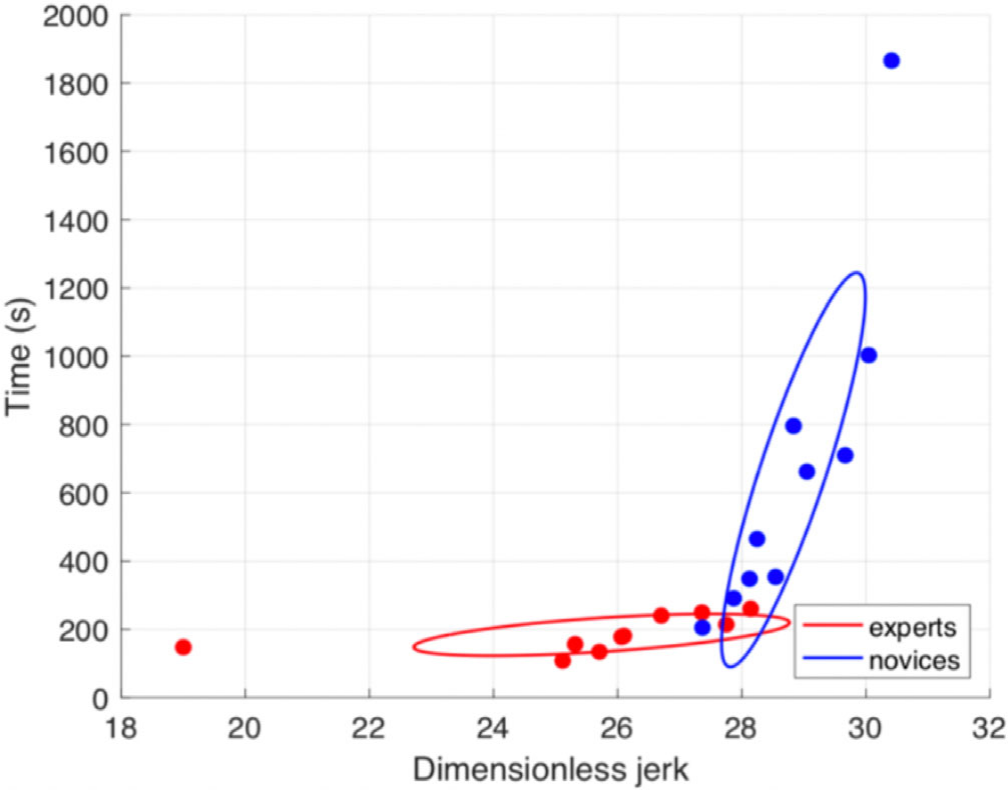
Dimensionless jerk plotted against time [Colour figure can be viewed at wileyonlinelibrary.com]

The mean DSJ for experienced operators was 19.26 SD 3.02 and 22.08 SD 1.05 for the novice group respectively (*p* = 0.01). The plotted DSJ against time to task completion is shown in Figure 3. The DSJ value and time taken are smaller for the experienced group and larger for the novice group. The covariance of the data is represented by the overlaid ellipses. Each ellipse contains results within an 95% confidence interval. The experienced and novice groups show little overlap and a distinct pattern of distribution, as seen in Figure 3.

## 4 DISCUSSION

Our results indicate that metrics such as time to completion, probe path distance travelled and DSJ show significant difference between novice and experienced ultrasonographers. When time and DSJ are considered together, the experienced group had lower DSJ, indicating that experienced operators had less unwanted and more purposeful movements than novices. Our results allow us to conclude that performance changes with experience can be quantified using objective measures.

The outliers in both the novice and expert groups are likely to represent the extremes of their respective groups. The expert outlier represents performance of obstetric biometry quickly and efficiently while the novice outlier represents an individual relatively inexperienced in performing these tasks. On the contrary it is the area where the two ellipses overlap that represent interesting areas for future research lies. The DSJ offers an objective measurement which could be combined with more traditional assessments such as trainee logbooks, patient feedback, audit of clinical images and trainer assessments such as OSATs to determine progression through training. As we reported in our literature review,^24^ it is worth noting that some clinicians will never achieve expert levels of performance despite daily use of ultrasound. We remind the reader that each ellipse contains results within an 95% confidence interval and, as such, outliers are to be expected. Our aim at this stage is not to try to move the outliers toward the mean but rather identify the likely extremes we might encounter if this methodology were to be used in a clinical setting and where a threshold between expert and novice performance might lie.

It is reassuring that our findings reflect the findings of similar studies describing the use of DSJ in MIS, where the path length of the arthroscope, the time taken to complete the task and the dimensionless squared jerk values were significantly different between experienced and novice groups. The authors expressed a view that the assessment of technical skills alone is unlikely to give an assessor a complete picture of clinical performance.^23^ In obstetric ultrasound, particularly when used as a point of care examination this is likely to be more pertinent. Further management of the pregnancy is likely to be influenced by the ultrasound findings. Technical skills which cannot be assessed by motion metrics, such as the interpretation of the obtained images and accurate placement of measurement callipers are combined with non‐technical skills such as communication with patients and colleagues, report writing and treatment planning to form the overall clinical competence. These cannot be assessed by motion metrics alone.^25^, ^26^ The current RCOG curriculum examines these through the use of Workplace Based Assessments. An objective measure of skill is likely to contribute to this assessment, rather than replace it.

### 4.1 Strengths and Limitations

This study used clinical ultrasound systems, a commercially available probe tracking system and the participants were reflective of the skills mix in many ultrasound departments. The use of clinical ultrasound machines and commercially available equipment makes the study reproduceable by other groups. The study is limited by small numbers in both groups and although completed in a clinical area, the ultrasound phantom was not able to simulate fetal movements, variations in Body Mass Index, or other challenges such as patient anxiety and distraction which may be associated with patient communication. Despite this, we have proposed a metric which is a first step in demonstrating performance differences between novice and experienced ultrasound operators. Repeating this experiment in patients rather than using a phantom may provide insight into how skills assessed in a simulated environment translate to clinical practice. Performing a scan in a clinical environment may induce stress or distractions which also impact on operator performance. Additional variables, such as gestational age, fetal position, maternal body habitus and fetal movements could affect operator performance.

## 5 CONCLUSION

DSJ may represent a metric by which training progress could be assessed. Alone, a single metric is unlikely to represent experienced skill, but could be used as a waypoint for trainees, signifying the transition from simulated learning to supervised practice. Integrating ultrasound simulation into training curricula and promoting self‐directed learning would give trainees the opportunity to contribute to the clinical service while learning a complex skill. In using DSJ, we propose a metric which gives trainees, and their trainers an objective measure of their progress over time which could be used in addition to other metrics that may, for example, be based on image data.

DSJ, alone cannot be assumed to equate to clinical competence, but objectively measures one of several technical and non‐technical behaviours required to perform obstetric ultrasound to a high standard. DSJ supports the idea that the process of performing an ultrasound scan is highly discriminatory between the novice and experienced operator. Further work is required to correlate this metric with tools traditionally used to evaluate training and standardised quality control gradings for images produced by the operator. These might include OSATs, as used by the RCOG and the ultrasound‐specific assessment OSAUS assessment, or the quality control framework adopted by the Fetal Medicine Foundation.

Our future work in this area will be to repeat this experiment in a clinical environment, adequately powered to show difference. Repeating the study in a clinical setting will allow us to understand if skills assessed in a simulated environment can be translated to clinical reality and how robust skills are when presented with the variability inherent in obstetric scanning owing to maternal habitus, stage of pregnancy, fetal presentation and position.

## CONFLICT OF INTEREST

Professor Jan Deprest is the Editor of the Fetal Medicine section of Prenatal Diagnosis.

## Data Availability

The data that support the findings of this study are available from the corresponding author, BD, upon reasonable request.
